# New Appliances and Things Medical

**Published:** 1896-02-08

**Authors:** 


### New Appliances and Things Medical.

[We shall be glad to receive, at our Office, 428, Strand, London, W.C., from the manufacturers, specimens of all new preparations and appliances, which may be brought out from time to time.]

### EXTRACT OF RED MARROW, LIQUOR THYROIDIN, &c.

(F. B. Benger and Co., Limited, Otter Works, Manchester.)

The above firm have already established for themselves a great name for their preparations, well known to the public and the profession as "Benger's Food," liquor pancreaticus, and liquor pepticus. We have lately had opportunity of testing some of their newer preparations, namely, peptonised chicken jelly, peptonised beef jelly, extract of red marrow, and liquor thyroidin. In the case of the two first-named it is not our purpose to enter into particulars, beyond stating that they have been appreciated by the patients on whom we have tried them, that they are carefully preserved in glass, and that they contain an overwhelming proportion of peptone. At the present moment we are using the extract of red marrow in a case of recurrent hæmorrhage from latent duodenal ulcer, and so far with most excellent results. It is in such cases as these, in which the system requires iron, and the stomach or bowel cannot stand crude salts of the metal, that the best results must be looked for. The extract appears to be well tolerated by the stomach, although all other food is administered by the bowel.

The liquor thyroidin is an extract of the fresh gland, each fluid ounce containing the active principle of two lobes. We prefer this form to the compressed tabloids, and even in preference to the extract prepared daily from the fresh gland. The difficulty in getting thyroid glands from the butcher, and their preparation by nurses, is a sufficient objection to this last method.

### FRAME FOOD.

(Frame Food Company, Limited, Lombard Road, Battersea, S.W.)

This food is already too well known to require detailed notice. We are prepared, however, to impart to it the hall mark of our approval as a highly valu able food for invalids and young children. Containing, as it does, a rich proportion of albuminoids and carbo-hydrates in a readily assimilable form, in addition to an unusually high percentage of phosphates, it is at once a food and a therapeutic agent. In its latter capacity it should be particularly useful in cases of rickets and deficient nerve energy.

